# Supplementary material for: Integration of Repeatomic and Cytogenetic Data on Satellite DNA for the Genome Analysis in the Genus Salvia (Lamiaceae)
Source: Plants (Basel). 2022 Aug 29;11(17):2244. doi: 10.3390/plants11172244 (PMC9460151; doi:10.3390/plants11172244)

Supplements:

Table S1. Characterization of the satDNAs revealed in repeatomes of *S. officinalis* and *S. sclarea*

Tandem Repeat/ Genome Proportion, %	Repeat Length, bp	Sequence
		Salvia officinalis
SO3/ 1.3	687	GAAATCCAACCTTTAGACCGCAGCGCTCGACCCACCATGTGCCAGGCTATTTTTGGCCACATCT<br>CATTCCCCGGGGGTAGGATCCGGTGGATCTTTACGTCGTCGGGGCTCATATACGAGGGGGAAG<br>CTATCACGGAGTTCTCACCCCGTTCTGAGCCGGCCCGAAAATAAGGATACCTTACGTGAGCTTC<br>GAATAGGCTATATTAAGCCATATTTCTATCGATACGAAAAATATCGACCGAGCCGAATGTCAA<br>ATCAAACCATCCCAAGTCCTTGGTCTATAAAAAAGGATCCTAACGCCGTGTTAATCACTCCAAA<br>CAGACCGATAAAGAAGGAGATATGGCCAAAACGGTGTTCGCGTTGAACGCAACCGTAGCACA<br>CTCCAATTTTTTGGCCATATCTCATTCGTTTCTAGTCTGCTCACAACGGTCTTTATACCGTTGGA<br>AAGCCACTTCAGAGGGCTACAATTTTTCTGAGTTCAGTTTCTCAAATCGAGCACTAAAATGGTC<br>CCGAAATCCAACTTTTTCCGACCACAGCGCACGATGCAACGTGTACCTGGCTGATTTCTTGGCC<br>ATATGTAATTCGTTGAGTGTCTTCTTCCAGCGGTCTTTTTACCGTTGGAAAGCTTACTCACAGGG<br>TTATGACTCTAGTACAGACAACTTTCGAATTGGGCCTTTTTCGAGCTTG
SO4/ 1.2	2884	CAAAAACTTCTCGGCTTTGGAAGGTGGGAAGTATTTCTCCAAGAACAATCGGCACATATCCTTC<br>CAAGTGGTGACCTTCTCTCGATCAAGCTCCTTGTACCATTCTCTAGCTTCTACACAACATCAACC<br>TATGTGCTTGATGGTTAGGAATCCCATAATTACCTCCAACACCTCCTATCATCCGGTTTAAACA<br>AGAAAAAGGCGGGCTAAGGCTCAAAGAGGCTAAATCAAAGGATAGTGGGTATGTGGGGTTAG<br>TGTATAGGATATGTGCCATGGTTGGTGAAAGTGGCGGTGACAAAGGATGGCCTAAGATCACTT<br>CCTAGTTCAAACACACACATGTGGTCTCGACGGAGAACCAAGGCAAGTTCTAGAATCCATATG<br>CATGTATGATATCCCTCAAGCAAGAAAATAGGCATAAAAATGCACCAAAGGCTCAAATCTCAC<br>GGGATAATCAAATAGTTCTCAAAATCAAAGGCAACTAATCCAAATTCATCATGCAATGCGTCA<br>CTTCGCCAAGGTTCCAATCATAATAAAGCCAAAATGCTATGAAATCTTATCAATCGCCGGCGCC<br>AAAATTTGATAGCACTTTTTCTAACACAAAATATGCAAGAAAATAGGGCAAGTATTATATATC<br>GTATCCACAGAGACTGAATATCGAAATTACAGCAAGTAATCCTAGTTGAGACTTCACCTCTCTC<br>CCCCCAAACTTATTCCACACAATACAAGGGGAATAAGGTTGGAAAAAGAAAAAGTGGCTATCT<br>TAAGCAACTTAATAAAAGAGAGAGAAAATAGATATAAAAATCAAAGATTAAAAGGTTGACCC<br>TAGGCAAGTGCTACTTTTAACCAAATTCACACAATTAACCTATTAATCTATTTACCAATTTTAAT<br>CCAAATTCATATGAAGGATCACACATGCAACCATAAAATTCCGCTAGGATAGATTATGATAGT<br>AGGCTAGTCTAAACCCTATCTCCGCTAGGTCTCAAGGGAAGTAAACTAGTTACCAAAAGCTCC<br>GACGTATTTAAATCCCTATGAACAAGTTTTCCTCGGGTAGGATAAACAAGTATCATATCATGCT<br>TCAATCATTCAATTATGCAGCATTGTAAATAGACATAAACTACACAACTAGGCATGCAAGACT<br>AGTGATCAATTAATCATGCATGAGAATAAGCTCATTGAAACATAGAATCCAAATTGGAAAGCA<br>TGACGAAGTCAAAAGGAATAATCCATACAAAGCTCAACTAGTTCATACCAACCCTAGGATCAT<br>GAGAAGTTTAGCTACTCATGATGAGGAGAAAGACATAAAGTAAATAATACTCCCCTGTATTGA<br>GCTAATGGTAGAAACTTGCATAAACTTGAAGTAAGCCAAGATCACACAACACCTTGGAGAAGG<br>TTTGAATCTAAAAACTCCAAAGAAAAACTCCCCGGATCCTTTTGCTTTGATGGGAGCTTGTTTT<br>GAAGTATGACACTACACTCCTCGGTGAGGTTGATCGTCTCAAACTTGCCCAATGCCACCGCATC<br>CTTGAGGAACTTCACATACCCCGGCCATGAGGGATGACGAGAAGAGGGGGTTTGAGGCCTATT<br>TATAGAGAGATGTCCAAGAACTTCTGCAACTTCTCGTCGTACTTCGGCTTGCGTAGGCATTGAG<br>GAAACGGAGCTCTGGGAGGAAAGTCTTGGGCTCAAAGTAGGTCTCGCCGCCAAGCCTCTCGGT<br>CTGGGTCTCGGTCGAGACCTGGACCGAGAGGGCACTGGGCGAGTTTTCGTTTTGGCTCGTTCTC<br>CCTCGTCCGGACTCCAAATCACGAACGGTTTTCGCCTACGGACTCCTATCGAGATGGACTTCAA<br>TCCTATGCATTCCAAACTCCTCAAATGATCCATCATGTTTCTGTAATTTTTCTAAACTTTGAGGC<br>TTGAATCCATTGGTGTAGTTTCTTTGGTCTCTTCGAGTCTTCTCTTCAACACTTGGGTGTCTTTTC<br>ATCTAAAAACCTACAAAACATACCAAAACAACCCAAAGCATAACAAAGCTCGACAAATGATA<br>AAACATATCCTAAACGAGCTAAAAGACCTTGAAAACCAACAAAACTAAAATTAGGTAAAATA<br>GGGCTCTATCAACTCCCCCAAACTTAACCATTGCTCGTCCCGAGCAAACAAGAAAAACAAAAC<br>AAA
SO44/0.37	170	GCGCGGCAATGCCGCTCGCACACCTTGTCCCTCCCGGTTTTCGTGTCCAAGCGTACCCAATGCT<br>CGGGTGTGCTAGCTTGTGTTTTGCTTCCGGAGATGCTACGCATCACCGGTGGCGGAGCGGCGGC<br>TTGTGCTCCCGGGCATTGTGTGCGTGGCGATGCGTGGTGTGT
SO46/ 0.36	135	CGGCACCAGCACGGGCGCCGCTCGACGGGACACGGCACCAGCACGGGCGGCCGCTCGACGGG<br>GCACGAGTCTCGCGCACGGGCGCGCGCGCATCGTCGGGGGCTGCCCGCGCAGGGGCGCCCGCT<br>CGACGTGACT
SO48/ 0.34	9722	CACACTAGATCACAAGCGGATAAGCATCACACACAACAAGATGTATATCATATAAGATCATAG<br>AGATAGGAAAAGATTCATGATAAACATCAAATAAGGTCCTCGAAAGATAGACATGCAGAAAG

ATTCACCAAGGATTCGAACCGAGAGAGAGAGAGAAGAAGAAAGGGTTATGCAGATGCCTACG
AGAGGGAGAAAGAAACAGACAATGTTGGCTGGCAATGTTAGGTCGAAGACAGTCAACATCCA
TACCCAACATTCATAACAGAGACTTGCCACCTTATGCAGATTTTTGTTCTTTAGAACTTTTGGGA
TATGATAAGATTTCAACTTCATTTTTCCTTGAGTGCATCGTCGCTGCGAACGCCGTGCCCCGAG
TACTTAGAAGTGAGCTTTTTGCACGATCCCTTTGAAGAGAATGTTTTCTGACCATTAAGCCTCA
CTCTTGTGCTACAGGTCTTATAACATCATTGTTGTGTTACACCAACATCTGCTTATGGCACAAGT
ACTAACACAAACGTCGGAGTACAAACATAACTTACTTCTCCCTGCAAAAGCCAGCAACATTAA
AACACATGCATATGCCTAAAAGAAAGAGGAAAAACAAACACAAGCAACAAAACAGAATGTTG
GGATCCAACAAAATCCTAAGTCCCAACATTCGAGCCACACACCTAGACTAGATGCAACACAGG
CCAAGAGACTTCCTAAGGTGAGAAAATCTCTCAAAATCCAAGGCTTTGGTGAAAATATCTGCT
ATCTGTTTTTCAGTTGGAACAAATTTAATGCAAATCAAGTTCTTCTCAACTAGATCTCTAATGA
AGTGATGCCTGATGTCAATGTGCTTAGTGCGAGAATGTTGTACAGGGTTTTTTGAAATATCAAT
AGCACTCATGTTGTCACATAACACAGTCAACATATCACTTTCCATACCATAGTCATCCAACATT
TGCCTAAGCCAAAGAAGTTGAGCACAGCAGCTTGCAGCAGAAATATACTCAGCTTCGGCAGTA
GAAAGGGAGACACAATTCTGTTTCTTGCATGACCATGACACGACATTGTTACCCAGAAAGTAA
CATCCTCCACTGGTGCTCTTTCTATCATCTGCATCACCTGCCCAATCAGCATCACTAAAACCAA
CAATGTTAGTGTTAGTGTCTTTAGAATACCACATCCCAAGTTCAGATGTTCCAGCAACATATCG
GATGATCCTCTTGACCGCTTTCAGATGGGATTCCTTAGGCATGGCTTGATAACGAGCACAGACC
CCTACACTGTACATTATGTCAGGTCTGCTGGCAGTAAGATACAGCAGACTTCCAATCATTCCAC
GGTACAATGTTGGGTCAACATTCGCACCAACATCATCTCTGCACAGCTTATCACTTGAACCCAT
GGGAGTCCTCATATGCTTGGCATTCTCCAGTCCAAACCTCTTGACAAGTCCTTTGGCATACTTA
CTTTGTGAGAAGAAAATGCCCCCTGGTGTTTGCTTAACTTGTAAACCAAGGAAGTAGTTGAGCT
CACCTACCATACTCATTTCAAATGTTGTTGACATTGCTTTCACAAATTCTTCAACAAGAGCAGA
GTTAGTAGCACCAAAAACAATGTCATCAACATATATCTGCGCAATTATGATGTTACTGCCATGC
TTCTTGATAAACAAAGTCTTGTCAACTTGACCTCTAGTGAAACCAAAATCAAGGAGAAACACT
GTAAGACGCTCATACCAGGCTCGAGGAGCTTGTTTAAGACCATACAAAGCTTTCTTCAGTTTAT
ACACATGCTCAGGTTTGTTAGAATCCTCAAAACCCTTAGGTTGTTCAACATAGGCCTCTTCAGA
TAACATTCCATTAAGGAAAGCACTCTTAACATCCATTTGTAGCAGAGAGATCCCTAGTTGACAT
GCAATGGCAAACAGGAGTCTAATGGATTCTATCCTAGCAACAGGAGCAAATGTTTCATCGAAG
TCAACCCCTTCCACCTGAGTGTATCCTTGAGCAACAAGTCTAGCCTTATTCCTAACAATGTTAC
CCTTTTCATCAGACTTGTTTTTGAAAATCCACTTGGTTCCAATAACATTCTTATCACATGGTCTA
GGCACAAGTTCCCACACATCATTGCGCACAAACTGATTTAATTCATCATGCATAGCATTCACCC
AGTCAACATCAGTCAATGCTTCTTTAACATTCTTAGGTTCAACAAGGGACATGTAACATGCAAA
CTCAACTTCTTCACAAACAACAGATAAACAAGCAATTCTTACCATCTCCCGGTAGTTTACCTGA
CCTTTCTTTCTAGTTTGCATCTGTCCAGAAGCATCCCCAATGATTTGGTCTTTGGGGTGATCTTT
TCTGATTCTTCCAGGTGGGTCTCGCCTCACTTGATCATGGAGAAAAGCACTGCCATCATCACCT
TCATCACTGTCAGAGGTATTGTCTCTGTGATCTTCAGTAGTGTCATCACTGGATGTGGGACCAA
CATCGGGTTGGACATGTTGTTCTGGTGTTTGTTCCGATGTTGATTGTACTTCCAACAGATCAGTA
ACATCATCTTCATCTGTTCTATCTGCAAGGCTCAACATATCATCGAAAACCACATTCACAGTCT
CTTGAATGGTTTTAGTCCGAAGATTATACACCCTATAAGCATGACTGTTTGCCGAGTAGCCAAG
GAACATCCCTTTATCACTTTTTGAGTCAAACTTGCTCAAATAATCCCTATCATTTAGGATGTAAC
AGACACAGCCAAACACATGAAAGTATTTGAGGTTAGGTTTTTTACCTTTCAAAATTTCATAGGG
AGTTAACATTGTACCCGGCCTCAAATAGACTCTGTTTATGATGTGACATGCAGTGTTAACAGCT
TCAGCCCACAATCTCTTGGAGAGGTTCTTGGTGTGTAACATTGCTCTCACCATCTCTTGGAGAG
TGCGATTTTTCCTCTCGGCAACACCATTCTGCTGTGGGGTTTTAGGAGCAGAGAACTCATGGAA
TATACCATTGTTAGCACAAAAATCAGAGAACAAAGAGTTCTCAAATTCCCTACCATGATCAGTT
CTAATCCTGACAACATTCTGTCCATATTGAGCACGAAAACGTTTGTACAGCTTTTTAAACATGA
AGAATGTTTCAGACTTTTCATGCATAAACTCAACCCAAGTATATCGGGAAAAATCATCAGCAC
ACACAAGAACATACCTCTTTCCACCTAGGCTTTCAACTTCAATGGGCCCCATGAGATCCATGTG
AAGTATTTCAAAACATCTAGAGGTACTGCATGTTGATAACATTGGATGTGAGGTTCGAACCTGT
TTTCCTTTCTGACATGGTCCACAAACCACTTCATTCTTGATCACCAGCTTAGGTAGACCTCGAAC
AATATCATTGGCTATCAGTTTCTGGAGATTCTTCAAGTTGACATGCCCTAATTTCTGATGCCAG
AGATCAACATCACACATCTTGGCTGTGTGGCAGCTGTTGTTGTTGTCAGTCTTGTAGCAGTTGT
CTGAGGATCTGCTTCCCACCATAATGCGCTTGTTGGAGTCATCAAACACTTCGCATATATTCTT
ATCGAACTTGACAAACATCCCATCATCACAGAGTTGGCTGATGCTTATCAAGTTTGCTTTCAGC
CCTTCAACATGATGAACATTCTTCAGCTTTGGGAAGTCTCTAACATTCAATGTTCCTTTCCCAAC
AATCTTGCCTTTGGCTCCTCCTCCGAAAGTAACATTTCCACCTTCTGTTGGAATGTAGTCACTCA
GGAACTCCTTGTTACCTGTCATGTGTCTAGAACAGCCACTATCAAAGTACCAGTTACATGAGAT
GTTAGCATTCAGAGAAGTGAACACCACATTGCATGTGTTAGAGAAGATTTTATCAGTCATAGC
AGAAATGTTTCTCAGAGAACTGCCAACATCAGACTGATAACAGTCAAACATCCCCTTGGCCGA
TGTTACATCAACATTGTTGTGCAACTTCTCACAGTAAGGTTTAATGTGTCCTGGTTCATGACAGT
AGTGACACACATAGGGTTTGCCTTTGTTCTTGGGCTTCTTCTTGAATTTGGGAAACTTGCCACCA
TTAGAGCCCATGAACACATTAGCATTCCCTTCACTCAAAAATACGGGAGCACTACAATTCTCAT
TCAACATGTTAGTGTTACCTGAATCAAAACCAAGGCCATGTCTGCCAAAAGATTTCTGAGATAA
AACACAGTTAAGATCAGAAGTTCCTTTGTTAAGGCGATCAAAAGTTTTTTCAGCCTTATACACT
TTACCTCTTAGCTCAAAAAGTTCAGTATCCTTTCTAGTGAGGAGGACTTCCAGCTTTAAGACAC
GATCTTTTAGGGTTTGATTCTCATCTAGGAGACCGAGAATGTCCCTTCTTTGCTCTTCACATTGA
GCCTCAACTTCATCATACAAATGTCTGATGTCATCAATAGCTTTGAGGGGATCAAACTCCTTTT
CTTCTGAGGCTGCAACACAACAAACATTCTCTGGAAAGATTTCAGCTTCATCAAGAAGAGTCAT
TTCATACCAGTTTGTTGTCGATGTTGGAAAGGTATGACCATCAATGTTGATCATGCAAAGATCT
TCCTCATCAATATTCTTGTCAGTGTCCATGACATCCTCATCTGAGAACTCTTCATTGTAATCCTC
TCCAATGTTGGCCATGACTTCAATCATTTCAGCCTCTGAGTCATCAACATTGTCATCAACATCGT
CTATTGTTGCAATCAGCAGCACCTTTTCTTCCTCTGAGTCAGAGTCATCGCTCAAGGAGGCAAC
ATGGCTGGCCTGTCTTTGCTTTTTCCTTGCCACTGTGGGACATTCATTAGCATAGTGCCCCATTC
CAGAACATCCCCTGCACTGAATACTGTCAGTTGAGTTGTCCTCAAACACAGGATTGTTGAATGT
TCGTCTAACATTGGGAATGTTAGAGGAGCTTGGGAATTTTCCAGTGGTGTTGGTGGCAGCCCTT
CTGGTGTTAGAATTTGGGACCGATTTTCCATTTCTGATAGATTTCACCAGATTATTGAACTTCTT
GGTCAACAGGGCAATGTTAAGATCTTCTTCATCATTGTCACATTCCAGACCTGTGTTCACAGTA

		TCAGCACTAACATTGGAAATCACATTAGAGCTTTCAGCCTGAAAGGCAACATGTTTGGACTTGT<br>AACCAGCCTTCTGTTGGTCGAGATTCATCTCAAAGGTAATCAATTTGCTCACAAGATCACCTAT<br>CCTCATGGTATTAACATCAATAGACTCCTCGATAGATGAGATTTTCATATTGTACCTTTCGGGA<br>AGAGATCTCATCATCTTACTTACCAATTTCTCATTAGCAATTGGTTCTCCAAGTCCAACAGCCTC<br>GTTTGCAATTTCACTTAACTTCTCATAGTATGATGGAATGGTTTCATCTTCTTGCATTCTCAACA<br>TCTCAAATCTCGTAGTCAACATCCTCATCCGTGTTGCCCTGACACTCGCTGATCCCTCACATTGT<br>TCCTGCAATATAAGCCAAGCATCTCGAGCCTCTGTGCAATTAGAGATCAGGCGAAAACATGGA<br>ACATCAACACAGATAAAAATAGCATTTAAAGCTCTAGCATTGAGATTAGAGATAAGTCTCTCTT<br>CAGCAGTCCACTGATTCTCATGTTTGTTGGTGACAACATCATTCACCGTTTCACGTGGAGGAGT<br>CCACCCATTTAACACAGCAAGCCACGCACGCTCATCAATGGATCTGATGTACATTGTCATGCGC<br>GACTTCCACAAAGAGTAGTTTTTCCCACTACTATCAAGTGCCGGAGGTCGAACGGAAGCGTTG<br>GAAGCGTCCATCAGTAGTAATTTCGCACACAGGAACGGTCTTAGTTCGTCAAGAACCTGCTCTT<br>GATGCCAATTGAAAGAATTCAGTGCCTGAATTACTACTTGTTAACACTGTGTGAATGTTAGTAG<br>AGTAATGTTACTAGCAATGTTAATAACATTCCAAGCAACATGCAGCGGAAATTAAAGAACACA<br>CAGGATAATTTTTGCGGGTTATGAATAACCCGCGTTGCCTCACGACAACCAAGCTCTATTGCAA<br>CTTAGAACAGATTACAAGAGTTACACCAGGAAACCTATCTACGGATTGCACTACCGCCTATGA<br>TAAAATCACCTAGTCTAACACTGCAAACTGAATGTTGCAACAAGCAAAAACAGTCGATACAAA<br>GATCGAACAAACAACCTGAACAGTGCAAGACGACTCGGTCTTCAACCCCACCCTTGCTTGCCT<br>AGCAATACCGTGGTCGATGTTTACAGATTGGCTGCCTAACACCGCCTCAGTTTAAGCGTGGTTG<br>AAGATGCGTTTCTTCACTGCGTTTCAGGAAAATGACGGAAAGGTTGGAGCGTATGATATCTCTC<br>AGAAAATAGCCTTCCAAATCCGTGCGTGAGAATGATCCATCTTGCAGCTTTTATAGAGGTGTAT<br>TTCGTGGTCAGCAAGGTCCACTTCCCACGTCCATAGCACTCCTACAACTGCTGCAAACCGCTCC<br>AGAAAACTGCTGCTCGAACTGCTGCAGAATGTTAGTGGAGATCATGCTCCTTAAAAATAGGAA<br>CTAACTCCCCATGATCTTGAATCCATGATTTAACTAATCTCCACTTATTCAAACGAGTCCCAACT<br>GCAATCCACGATTATTAGAGTAACAAAGAATATAGGAAACGTGGAGGAAGGTAAAGATTACA<br>TATATACAGTTACGTTTGGAGAGTCGAAGTTCAGCTTCAAGGTAGACTCCAGGAATGTTGACTG<br>TACCCAGATGTTACCAACATCGGAATGTTACTAACATGCTATCCAACATTGCTGGAGTCAACAT<br>TGACTCTAACAATCTCCCCCTTAATCTTAGCCTTCCTGCAAATTTCTGCAACAAAAACAAACA<br>AAGAAAACCAAAAACTATTTACAGAAAAAGAATTCTATGTTACAGGTTAAGATCGAAGGAAA<br>GTGTGGAAACTCCCCCTCAATCAAACAAGAGGTAAAACCTCAAAACATTCAGAGAGAAATGTC<br>AGACTAACACTCCCCCTGAAACAAACACGGGAGAGACTAAAACTCCCCCTAAACCAATGAAGG<br>TCGGCCGAAGGCCAAGACCTTCAATTCGATGTTCTTCGAACTGACATGTACCTGAAAAGAAAC<br>AAGCATAAACAACTAGCAACATAAACATTCACCATTCACACCATGACAAGTGAAAGGAAATCA<br>TGCATCATTGCCAAAAATCAAGGTGTCCATACACCAAAACGATCAAGATGTCCATGCATCAAA<br>AACCAACTAAAGGAACCAAAGTGTCTAGCACTTAAACAACAACCAAATCGAATAAAATGTTTT<br>TGGAGCCAACATCAAAAACAGCAGCAAGCAGGCGCGGCGCAGAAATAGCTACTCCCCCTTTTT<br>TGTTGAAGGAAAGAGAAACGGCAGGACCTTCCCGAACTCATCAGCAGTAGCATCAACTCGAGC<br>AGAGAGGGAAATGCACATCTTCTGGACATATTCAGCAAATTTGGCAGTTGAGTCCGACAGTTT<br>CTGAATGTCACCCTTCAATTTCTTGAAATTCTTCATTGCAGCTTTGAGATCATCAGGGTCATTT<br>GAACTTCCAAGAAGTCGGTAGCAACATCCTCTTCCTCCATAGGAGCTTTCCCTTTATCAGCTGC<br>ACGAGAGGTACCTGCATCAACATTAGTAGCAGAGGGGGCAGAAGAACCAGACTCTGAGGATG<br>TGTTGGCAACATTGCTTCCAACATCCTTCCTTTTCTTCGATCCTTCTTCTACTTCCTTCATCAGCA<br>CATCGAAGGAGTCTTCCTTCTCTTCTTCACCAACATCCTCTGTCTCAACATCAGGAGTGTATCGA<br>CGCTTGGCTTTAGGAGGACTGTAGGGGAGGTCCTTCACTCTATCTGAGGATAAGATAGTCTTCG<br>CAAACTCCAGAGTGTTGCTCTCCTCAACCAACATTGTACCCTTGTCAGCCCGGCGTAGTCCTTG<br>AGATCTCAGGAGGTTGTAGATCAGGCTCGGGAAAGGGAGAGTGTTGGACCCCGACGACTTGAA<br>TGCTGCACGTGTTATGAAGGCAAAAACCAACTTACCAAAGTTGAAGTCAAGAGACTTACCAAC<br>TTTATAGATAAATTCTGCTTGATCACGAGTCAAGGTAGTTGAGTTTTTGCTCGGCAACCAGTTA<br>TACACACAAATCTTGTGCAATGTAGCATAGAGTCGAGAGAGTGAGGTAGCAGCAGTTTGAGGC<br>CTCCACACATTCAGTCTGCCTCCAGTGATAGTTTTCATCATTTCCTTCTCCTTCACTGTTGACTC<br>AACATCAACATCTTCAAAATAAAACAGCTTGTTGATGGCCGAAGGAGAGCAATCAAACACTTG<br>ACCCCGCACATAAACCTTCCCAAATTTCTTGGAGGTGGTATCAGAAACCTCCTTCAAAAGATTA<br>GAGTAGAACTCCCTAACAACATCCTCATCATAGGGATAGATGTTAACCACAGTGCCCCACATCT<br>TCCTATCTTTTAACAACACATCAAGACCATAATTCTTGAAAAACTTAGCATCAGCCTTCCTCTC<br>ATTAATAAAGTTCCTATCTAACATACTCTTGTGAATAGTCTTAGCCTCACGAGAGTAAAAGCGG<br>TTAGAATAAGCCTTACCAACCTCAATGTCAGAATCGGAATCCTCTTCAGAATTGCTTCCTTGAG<br>GTGATTGAGAGGGAGATGCTCCACGAGATGATGTTGCCTTGCCACGAACCTTGGTGGAGATAG<br>TCGGTATGGGGAGCTCCTTTCGTCCAGAGATGTTGGGCTTCTTGGATTTCGATGTTGTAGTAAC<br>ATCACCTTTACTGCGCTTGGGCAGATTTAAATTTGGCGGCACCGGGAGAGAGGATTTTGACCTA<br>GTGCGACGAGCTGCACCACCAGAGAGGGTGATGACCTTCTCCTTCCCTTTTCTCGTGCCCTCCT<br>TTTCGCCGACAATCTGGATGTCGTCGTCAGCCACATCCTCTGTTTCTTCAACATCAGCCTTCGCC<br>GGAGACGCAGGAGACTGATCAACATTCTCCTTGTGCTCCCCCTGTTCATCTTCCATGGTGGTAT<br>CAACATCTGCCTTTGAAGGGGAGCGAATCGACTCTTGGCTGTCGGCCGAGGCGAAAGGAACAA<br>TCTGGGTCTTCACCGGAGTTTCCTCTCCTCCGTCCGTTTTCTGCTTCTTTGCGGCCGTGTCTTCCT<br>CTCCAACATCGGGAGAGACCCGCTTCTTCGCCGCCTTTCTTTCCTCCGCCTTTGCGGTGTAGAG<br>AACATTCAAAGGAATGTTGTCTGGGTCCTTTGCAATATCTTCTGGGAGGATCATACTGATTGGC<br>TCGACATTCAGGAGAGGATCAGACTGTGGATCGGCGGTGTTGGACGGAGAATCGGATGGTTC<br>ATTTTGGACAATGTTGGGCGAGAGGATTGAGGGAGAGATGTGTTTTGGGCAGATTCGAAAATA<br>GGGTTTGTGATGGGAGAGGAAGTCTGTTCGCCTTCTTTAAGAGATGATAAATGCTGGGCAGCG<br>GCTTCTGGGGTTCCGAACTTCTTCACAAAGCTTTGAAAGAGATTGGCCAAGTCCTTATCCATGT<br>TGATTCAACACGCTGCACAAACACTTTAACAACACAAGAAGCTAGAGAGGGGTTTAGTGAAAT<br>AACAGAGATATGAAGCACATATATATACCCCAACCGGCAGAGCCCAAGCCCACACAAACACA<br>AGGAAAAAACCCTAAAAAA
SO97/0.075	721	ATTGCGTCGCTTCAGCCATTCTTTTTCCCAAAAATTGTTTTGGCCATATCTCCTTCGATATACAT<br>CAGATTAGGACAATTAACGCGGCGTTGGATTCCTTATTCGGAGCCCTACGATTGGGGATGGTTG<br>GATTTGACATCACTCTCGGTCGAGATTTTCGATATTTTCGAGCCATCGGTCGTAGAGGCTCATA<br>ATCTTAGCGAATCCCATTTTTTTAGCCCCGCTCCGAAAGGGGCAAGAAGCACGTCATAGCTTCA

		CCCTCTTATATGATCCTTGACAACGTACGATTCCACCGGATACTACCACAAACGAATGAGATGT<br>GGCCAAAAAACAGTGAGAAACACGGTGGGTTGAACGCTACGATTGGAAATAACCAATTTCGAC<br>ACCCCAAAAGGGATCCATTAGAATAATTGTCTAAGCCAAAGTCGTATCCCTCTGAGTAATCCTT<br>CCAACGGCGTGAGGACCAATGAGATACGACAACAAATGTGTGAGATATGGCTAAAAAGGCGA<br>GATAAATAGTGGGTCACATTTTATGATCAGAAAAACTAGATTTCGAGACCTCGAAAATACCCG<br>ATTCGAAATATTGTGTCCAGAAAAGTTGTAGCCCTCTGAGTAAGCTTTCCAACGGTATAAAGAC<br>CGCTGTGAATGGACCACAAACGAATGAGATATGGCCAAAATACGGGCATGTACACCGGTTGCC<br>TTCTTTTTCCTTTTTTTTTG
SO107/ 0.058	362	TTGGAGGATCCGCCAAAAATACGGAAAATTTCGACCGAGCCAAATGTCGAATCCGACCCTCCC<br>AGACCTTAGGGCTATGCATAAGGATCACGAGGATGCGTTATGGGCCCCGAGACGGCCTACGAA<br>GAAGGAGATATGGCCAAAACAGTGTTCTGGCAAAGGCCAATCGGAGCGACGCCGGCGTTTTAG<br>CCATATCTCAATCGTTTGTGGTCCTGTCGCAGCGGTCTATATACCGTTGGAAAGCCAATTCAGA<br>GAGCTACGACTTTGCTGGATGGTATTTGGCAAAACGGGATCTGCATCGGCCCCAAAAATCGAC<br>TTGAAGAGCTTGCCGTGAAAATGAGATTTTCTCAAAGGGGTACCCC
SO108/ 0.056	352	CAGAAATTTTTCGTATTTTTGGGCCCTTTGCTATTCGGTCGACCCAAGCAAATATTCTCATTTTT<br>ACGAAAGGCTTTTCGAGACCCGAAAAGGGTCCGAATCGAAAAATTGTCTTCACCAAAGTCGTA<br>GCCCTCTGAATTATCTTTCCAACAGCATAAAGATGACCGTGACAGGACCACAAGCGAATGAGA<br>TATGGCCAATAAACAGGCGTATTCTTCAATTACCTTTCACCCGAACACTGTTTTGACCATATCTC<br>CTTAGATATGGGCTGTCTTGGGCCAATTAACACGGCCTAGTGATCCTTATTCGGTGCCCTACGG<br>CTTGGTAGAGTCAGATTTCACATTTGGCTCGG
SO113/ 0.052	178	CAAATCCTAAACCCTAATTGTAGATTTTAACCAAATTGTCTTATTGGTATGCTTTATTTGTCTCA<br>TTTTGACTTCATATTAGCCTCATTTGAGACTTCATATTTGTCTCGTTTATAGAAATTGTGGACAC<br>TTGTACCGTAGGAGTATATATGCTTCTCTAAGCCCTAAACCCTAATTT
SO145/ 0.032	1564	TTCTTTTCAGGCATGGAACTGCAGCTATGAGTTCTTGAGCTAATGAGCTAGGCTGCTCTGAGCT<br>AACGGCCCGTCAATTCCTGAGTCATCGATGGAATTGAATAAGGGCCTACCGATCCTGTTGCTAG<br>AAGCTAATGAGCTAATGTAGCTACCCGCTCCTACCATTCCGAAGGCATCCAAGGAATGGAATA<br>AGGAGCGACCTTCTCTCTTCTATGAAGCTATGAGTTATTGTAGCTTCTTGTTGTTTATGAGCTCT<br>TAGCTACTCCTTTCCTTTGTCTTATCTTATCTTGCTTTCTATGGCTTTAGAGTTGCTATTAAAGAG<br>CTATAAGAGGCAGCTAAGGACTTGTTTGTTTTGAACTTCTTTAGCTGCTAACTATCTCTATAGCC<br>CGGACCATGAACACAATCTTGAACTCGGACTTGTGACAACTGACAACGAGGGCTTCTGCTTCTC<br>TCTGTTTTCCTCCTGGGTACTTGACTAGAACTTGAGAGTCAGGATTTGAAGACTCTCAAGGACG<br>AGGAAAGCAGCTCAGTATGAACTACAGTGATTCATACCGTCAGAACAAACTCGAACAAGAGA<br>GGGGATATCCACTGATATGCCCGAACGATAGAGCTCCCGAACTTGTCCGTACATCGAACGAAC<br>CCTCGGATAGGAAACTCCTTGTTTTCTTGTTCTTTTCACAAACAAACATGTAACTCTTTCTCTTC<br>CTTACTGAAAGCCAGAGAGTGACCTACTAGTTGTAGGGCACGATCGACTTAGAGTTGCTAAGA<br>GCGCCCCCCAGTCCCGTACACAAAAAGGGAGGGATGGGGGGTGCGACTCAGAGTGAAGAAAG<br>GAATGGAACCAAGTGATGGACTGTCAGGTGACAGGCCAGAACGATATGGTACCAGGAGTTAC<br>GCGCGCGCACGAGGAGTTCCAAAAATGGCTTTCCTCGATAAACTCTGTTGTTTTATTGCGCGCC<br>CTTACAAGTTGTTTGTTAGCTTGTTCTTATTGCTGCTTTCTAGTCAAGTACCTGTACCAGTATGG<br>AGCCAACGTCGATCGCTTTCAGCTTGGGAACCTCTATTTTCGCTCGATGAGAGGATTCCCGCTA<br>TCATCTAGGGCCTTTATGGGTTTTGAAAGCAAGCCACCCCAGTTCATAGCACACTACTGCCTTC<br>TTGTCCCTTCGTTCATAGCTAGGAGTTCATAGGTAAACCCAAGATAGTCTCACTTCCAGCAGTA<br>TTCAATCGACTGATGCTACACTTGCTAACCATCTCACTAACTAGACCGAAGGGGTTCAGTAGTC<br>ATCTACGACATTTCCAATTGAACTCTAACTCCTCATTTGTTGCTTGTTTTCTTTTTAAAACAGAC<br>ATGTAAACTTGTCTCTCATGAGCTGTTTGCGAGTGAGTGACCCATCGAAATGGGGCACGAACG<br>CCTAAGAGTCCTCTTTACTGAGCTCCTTGTTTACAGTTGTTACTGAGTTCCTGTAGCTATGAGCT<br>AAGGAAGCTAGCTGCTAGAACGCGCTAAAACACGAGTTTAACAGGCCTCACGATAGATTGTAT<br>TCCAAGATCTGAAACCTTGTCTTTTAAGCTC
SO202/ 0.021	181	GGAAATAGTTACCAAGTACATCACCATAAGTATTGGAAATTTCCACGGTAGGCGTTACCGTGG<br>AAATTATTCTAAGTGTAGAAATTTTCACGAAACGAGTTTTCGTGGACATAACCATTAGTATTAG<br>AGATTTTCATGCTAGGAATTATGCTAGAAAGCACTGTATGTGTAGAAATTCAAC
SO342/ 0.01	133	ATATAGAATATAATATATGGAGTTTCATTATATTAATATACTCTATCATATAATATAAGTGAAT<br>ACATTATACTTATTCATAATATTAAGATATGAGACTATACTTTAGTCTTTTTCTTATTACTTTATT<br>TAA

Salvia sclarea

SS1 /1.9	362	GAAAGTCGTAGCCCTCTGAACTGGCTTTCCAACGATATGCTGACCGCTGCGGGGCGACCTCGTA<br>CCAATGAGATATGGCCGAAACGCCGGCGTCGCTCCATTCGGCCTCTACCGGAACACTGTTTTGG<br>CCATATCTCCTTCCCCGTAGGTCGTCTCGGGGCCCATAACGCGTCCATTTGATCCTTATGTATAG<br>CACTAGGCCATGGCAGGGTCGAATCCGACATCTGGCTAGGTCGAAATTTTCCGTATTTTCGGCC<br>AAACCTCAAAGGGGGTACCCTTGGGGAAAATCTCATTTTCAAGGAGAGCTATTCAGGTCGAAT<br>TTCAGGGCCCTTGCAGCCCCCGTCTCGCAAAAGTCCATCCAG
SS8 /1.3	158	GAAGATTAGAGTGCGAAGGAACGGCCTCGTTCTCGTCGGAATTGCTCCGAAATCGTGGTCTCTC<br>GTTGAAGGCCTAACTACCGGAGTTGCCGTTTTTCTTGAGAATTGCTCGCTTTTTCGACCAAAGC<br>GCACCAAACATACCTTTATTATGATGTGTC

SS123/0.056	170	TTTTTTCGACCAAAGCCCACCACACACACCCTTATTGTGACGTGTCCCGCGTAAAAGTGTCGAA GAACGGCATCGTTGTCGTCGAAATTGGTTCGAAATCGAGGTTGTTCCGTAGGAAGCTCATTGCT AGCTTAACTCCGTGAATCCTCGTTTTTCTTTAGAATCGGCTG
SS337/0.0098	397	TTGGGTGGTAACGGCGGTGCAAGATGTACGGATTGTCGTTCGAGGGGAGGATTGTTGGGGTTG GCAAACCCATCCCACATCGGAAGATAGAAGAAGGAATAACTTCTATATAATAAAGTCCACCAC TAATTAGTATGAGGCCTTTTGGGAAGGAGCCCAAAAACAAATCCGTGCGGGCTCGGCCCAAAG CGGACAATATCATACTAATGACGGAATGGTGGCGGTGGCAGTCCCTAACAGTGGTATCAGAGC CGATGGTTCGGCTGGGCCCAATATGTGGGCGGGTGGAGACTGCTTTCCCGATGATGGAGGTAA ACCCCCAGTGAGCTCACGATTGAATCCGGTGGTTCCTTGTGTCGAAAGTCTTCCTGATAGTGTG GTCAGGCGGCGAGTCCCG

Table S2. BLAST homology and FISH chromosome mapping of the satDNAs revealed in repeatomes of *S. officinalis* and *S. sclarea*.

Tandem Repeat	Blast Homology (between the Revealed Tandem Repeats)	BLAST Homology (Available NCBI Data)	FISH Chromosome Localization
		Salvia officinalis	
SO3	69% of identity with SO97; 74% of identity with SO107; 71% of identity with SO108, 69.4% of identity with SS1	no	A large cluster was localized in the subtelomeric region of the long arm of chromosome pair 4
SO4		*Theobroma cacao* genome assembly, chromosome: II, LT594789.1	A large cluster was localized in the subtelomeric region of the long arm of one or two homologs of chromosome pair 5
SO44		no	Small clusters were localized in the pericentromeric region of all chromosomes;
SO46		no	In the NOR region of chromosome pair 3
SO48		Putative LTR elements; *Lotus japonicus* B-129 DNA, chromosome 2, AP022630.1; *Quercus robur* genome assembly, chromosome 9, OW028772.1	A large cluster was localized in the subtelomeric region of the long arm of chromosome pair 4
SO97	69% of identity with SO3; 86,5% of identity with SO107; 77% of identity with SS1	*Ipomoea trifida* cultivar NCNSP0306 chromosome 13 CP025656.199;	A large cluster was localized in the subtelomeric region of the long arm of chromosome pair 4
SO107	74% of identity with SO3; 86,5% of identity with SO97; 80% of identity with SO108; 83.7% of identity with SS1	no	A large cluster was localized in the subtelomeric region of the long arm of chromosome pair 4, and small clusters were detected in the subtelomeric regions of both arms
SO108	71% of identity with SO3; 80% of identity with SO107	*Ipomoea trifida* cultivar NCNSP0306 chromosome 15 CP025658.1; *Jasminum sambac* cultivar Hutoumoli linkage group Lg13 CP073644.1	A large cluster was localized in the subtelomeric region of the long arm of chromosome pair 4
SO113		no	Small clusters were localized in the pericentromeric region of all chtomosomes
SO145		no	A large cluster was localized in the subtelomeric region of the long arm of chromosome pair 4
SO202		no	A large cluster was localized in the subtelomeric region of the long arm of

			chromosome pair 4, and a minor site was detected in the subtelomeric region of the short arm of one homolog of chromosome pair 6
SO342		no	Dispersed hybridization signals were distributed along all chromosomes
		Salvia sclarea	
SS1	83.7% of identity with SO107; 77% of identity with SO97; 69.4% of identity with SO3	no	Small clusters were localized in the subtelomeric regions of both arms of all chromosomes. In *S. officinalis*, a large cluster was localized in the subtelomeric region of the long arm of chromosome pair 4
SS8		no	Small polymorphic clusters were localized in the subtelomeric regions of both arms (except chromosome pair 5 and 11) and also in the pericentromeric region of chromosomes
SS123		no	Small clusters were localized in the pericentromeric region of all chromosomes
SS337		*Arabidopsis arenosa* genome assembly, chromosome: 4-8 LR999455.1	Small clusters were localized in the subtelomeric regions of both arms of all chromosomes

Table S3. List of the oligonucleotide FISH probes.

Tandem Repeat	Oligo FISH probe sequence
SO3	GAAGGAGATA GGCCAAAAC GGTGTTCGCG TTGAACGCAA CCGTAGCACA CTCCAA
SO4	AGAAAAACTC CCCGGATCCT TTTGCTTTGA TGGGAGCTTG TTTTGAAGTA TGACA
SO44	TTTGCTT CCGGAGATGC TACGCATCAC CGGTGGCGGA GCGGCGGCTT
SO46	CGCGCATCGT CGGGGGCTGC CCGCGCAGGG GCG
SO48	ATCCAAGGCT TTGGTGAAAA TATCTGCTAT CTGTTTTTCA GTTGGAACAA ATTTAATGCA
SO97	AAAGTTGTAG CCCTCTGAGT AAGCTTTCCA ACGGTATAAA GACCGCTGTG AATGGACCA
SO107	TTGGAGGATC CGCCAAAAAT ACGGAAAATT TCGACCGAGC CAAATGTCGA ATCCGACCCT
SO108	CCTTTCA CCCGAACACT GTTTTGACCA TATCTCCTTA GATATGGGCT GTCTTGGGCC
SO113	TTGTGGACAC TTGTACCGTA GGAGTATATA TGCTTCTCTA AGCCCTAAAC
SO145	TCGAACAAGA GAGGGGATAT CCACTGATAT GCCCGAACGA TAGAG
SO202	GGAAATAGTT ACCAAGTACA TCACCATAAG TATTGGAAAT TTCCACGGTA GGCGTTACCG
SO342	ATATAGAATA TAATATATGG AGTTTCATTA TATTAATATA CTCTATCATA
SS1	GTCGTAGCCC TCTGAACTGG CTTTCCAACG ATATGCTGAC CGCTGC
SS8	GAATTGCTCG CTTTTTCGAC CAAAGCGCAC CAAACATACC TTTATTATGA TGT
SS123	TCCGTAGGAA GCTCATTGCT AGCTTAACTC CGTGAATCCT CGTTTTTCTT TA

SS337	AGGGGAGGAT TGTTGGGGTT GGCAAACCCA TCCCACATCG GAAGATAGAA GAAGGAA

Figure S1. The raw images from Figure 5.

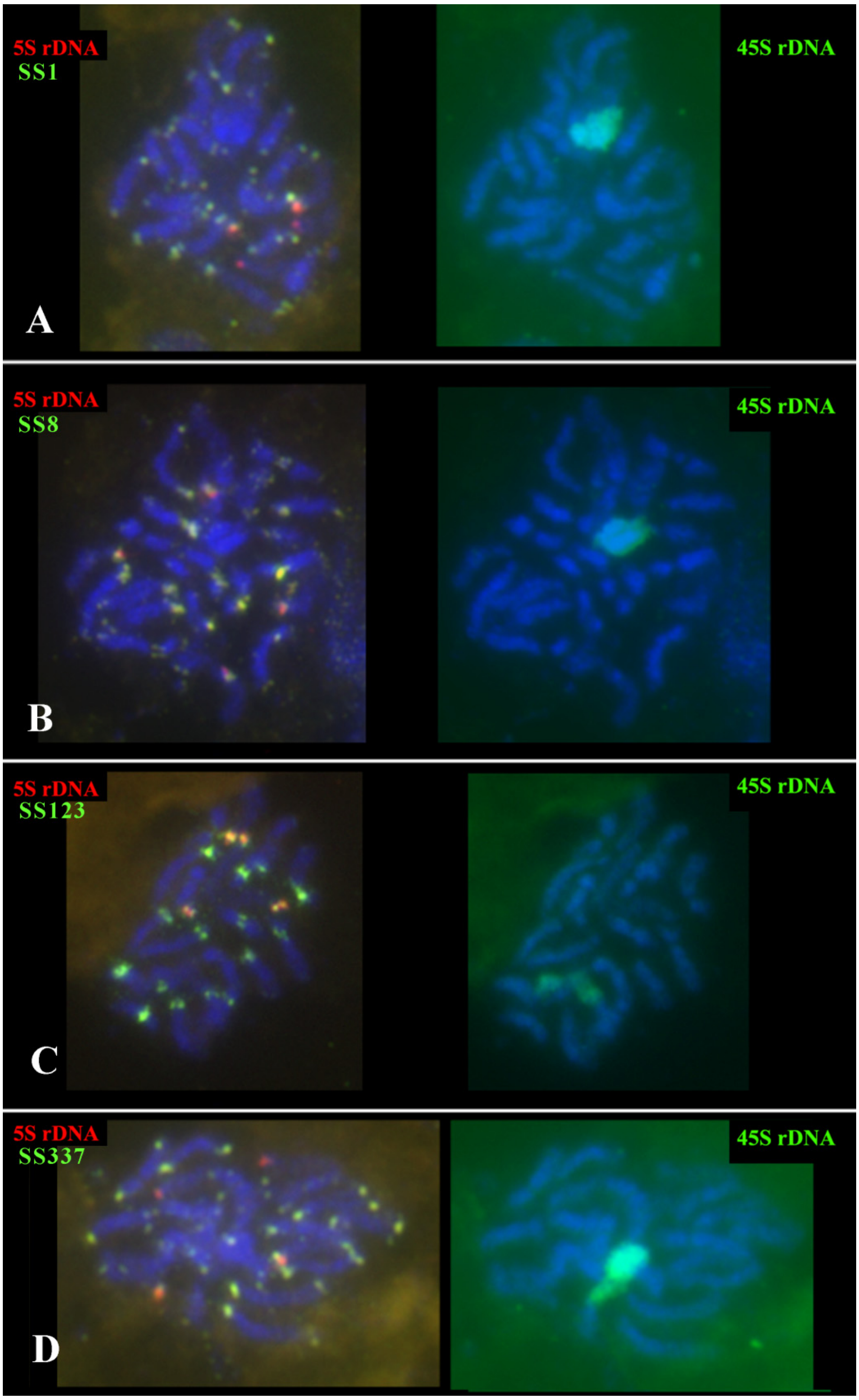

Supplement: Supplementary file 1 [file plants-11-02244-s001.zip › plants-1865094-supplementary.pdf]
